# Impact of Tunisian Fermented Zgougou and Related Lactobacilli on Caco‐2 Cell Barrier Integrity: Upregulation of Key Tight Junction Proteins

**DOI:** 10.1002/fsn3.72387

**Published:** 2026-09-27

**Authors:** Jihen Missaoui, Marina Liso, Mohamed Lataoui, Rosangela Limongelli, Fabio Minervini, Lotfi Achour, Maria De Angelis

**Affiliations:** ^1^ Department of Soil, Plant and Food Sciences (Di.S.S.P.A) University of Bari Aldo Moro Bari Italy; ^2^ Bio‐Resources: Integrative Biology & Valorization (BIOLIVAL), Higher Institute of Biotechnology of Monastir University of Monastir Monastir Tunisia; ^3^ Functional Gastrointestinal Disorders Research Group National Institute of Gastroenterology IRCCS “S. de Bellis” Castellana Grotte Italy

**Keywords:** fermentation, lactobacilli, tight junction proteins, transepithelial electrical resistance, tumor necrosis factor α, zgougou

## Abstract

The intestinal barrier is a critical regulator of gut health, often compromised by dysbiosis and inflammation. Given the rising interest in fermented plant‐based foods and probiotics as adjuvants in maintaining gut health, this study explored the biological activity of zgougou juice, comparing spontaneous fermentation (J) with driven fermentation using two lactobacilli with probiotic traits (namely *Lactiplantibacillus paraplantarum* A1 and *Lactiplantibacillus plantarum* A2), as well as the effects of cell biomass and Cell‐Free Supernatants (CFS) obtained from those lactobacilli. Using a Caco‐2 cell monolayer model, we assessed barrier integrity via Transepithelial Electrical Resistance (TEER) and gene expression of Tight Junction Proteins (TJP) and the cytokine Tumor Necrosis Factor α (TNFα). Treatment of Caco‐2 monolayer with zgougou juice fermented with lactobacilli significantly reinforced the intestinal barrier, with TEER values reaching 690–963.33 Ω·cm^2^. Furthermore, it upregulated TJP expression, outperforming bacterial biomass and their CFS. These findings would suggest that zgougou juice improves intestinal barrier, though further in vivo and clinical trials are necessary to claim any benefits for human health.

## Graphical abstract

Fermented zgougou and related Lactobacilli improve barrier integrity of Caco‐2 cells.

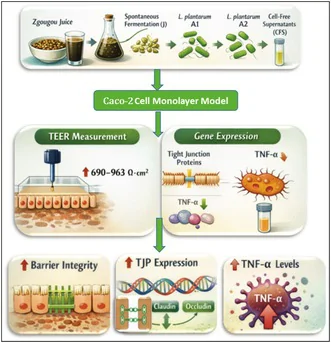

**Abbreviations**
Caco‐2 cell: Human Caucasian Colon Adenocarcinoma Cells
CFS: cell‐free supernatant
CLDN2: Claudin‐2
GAPDH: glyceraldehyde‐3‐phosphate dehydrogenase
IBD: Inflammatory Bowel Disease
J: Zgougou Juice Obtained Upon Spontaneous Fermentation
J(A1 + A2): Zgougou Juice Obtained Upon Driven Fermentation With Probiotic Lactobacilli
LAB: Lactic Acid Bacteria
LCN2: Lipocalin‐2
LPS: *Escherchia coli* Lipo Poly Saccharide
OCLN: Occludin
RT‐qPCR: real time‐polymerase chain reaction
TEER: trans epithelial electrical resistance
TJP: tight junction proteins
TJs: tight junctions
TNFα: tumor necrosis factor alpha

## 1 Introduction

Aleppo pine ( *Pinus halepensis* Mill) seeds represent the main ingredient for a Tunisian fermented food known as “Assidet zgougou.” Its putative health benefits are anecdotally known (Botella and Diez 2011; Schiller 2014; Kadri et al. 2015; Zaroual et al. 2019; Ait Atmane et al. 2021). These health benefits could be linked to its content in phytochemicals, especially to phenolic compounds with analgesic, anti‐inflammatory, and antioxidant capacities (Minervini et al. 2020; Ait Atmane et al. 2024). Besides phytochemicals, lactic acid fermentation characterizing the production of zgougou juice may result in antimicrobial and antioxidant activities attributable to the microorganisms that drive fermentation (Tukan et al. 2013; Missaoui et al. 2019; Minervini et al. 2020). In turn, such activities can positively influence the gut microbiota composition and function, leading to improved gut barrier function (Mosiej et al. 2025).

Intestinal epithelial barrier, having a pivotal role in maintaining the human body in good health, results from complex interactions that involve biochemical, immunological, microbial, and structural components (Han et al. 2023; Mosiej et al. 2025). Besides the mucous layer and gut microbiota, Tight Junction Proteins (TJP) of intestinal epithelium are of utmost importance for its “gate and fence” function. In fact, TJP are typically located in the *zona occludens* between endothelial and epithelial cells. They are essential for controlling cell permeability to water, ions and other solutes that move through the paracellular route, by creating selectively permeable cellular barriers that divide the body's apical (luminal) and basolateral (abluminal) sides. This allows the tight junction to control the transport processes and preserve homeostasis (Suzuki 2020; Paradis et al. 2021; Zhang et al. 2022). Expression of TJP, such as occludin and claudins, is highly tissue‐specific, dynamic, and finely regulated. Occludin (OCLN), the first identified TJP, stabilizes and provides structural integrity to the tight junctions existing between the intestinal epithelial cells (Zhang et al. 2022). When over‐synthesized, it limits the cytokine‐induced increase of gut permeability and its expression patterns change during development or in disease states like cancer (Wang et al. 2021). Different claudin isoforms (e.g., 1, 3, 5, 11) confer unique fence properties to organs like kidney, lung, gut, as well as to blood–brain barrier. In particular, Claudin‐2 (CLDN2) is associated with improved permeability of intestinal epithelium (Campbell et al. 2017; Zhang et al. 2022). *Lactiplantibacillus plantarum* considerably enhances the intestinal barrier and prevents “leaky gut” through improvement of tight junctions (TJs). Certain strains of this species increase the production and appropriate localization of TJP, such as *Zonula occludens*‐1 (ZO‐1), occludin, and claudin‐1, by increasing the transcription level of their corresponding genes; this, in turn, reduces intestinal permeability and guards against inflammation (Devi et al. 2024). Tumor Necrosis Factor‐α (TNFα) is an inflammatory cytokine that regulates integrity of TJs through internalization of OCLN, which elevates gut permeability. Lipocalin‐2 (LCN2) is a further protein component of the intestinal epithelial barrier, with antibacterial activity, which seems to be induced by inflammatory stimuli. LCN2 is a crucial iron regulator and bacteriostatic factor in innate immunity, exhibiting complex roles in a variety of diseases like Inflammatory Bowel Disease (IBD), cancer, and metabolic disorders that frequently involve gut microbiota in a biunivocal cause‐effect relationship (Saha et al. 2017; Sahu et al. 2023). Some previous reports demonstrated that bacteria of the *L. plantarum* group interacts with LCN2 mainly through the immune system. Certain strains of *L. plantarum* can induce macrophages to secrete LCN2 that, besides limiting iron nutrition by undesired bacteria, boosts anti‐tumor immunity or influences inflammation (Prasetyo et al. 2020; Sharma et al. 2024).

Oxidative stress and several pathological conditions may damage intestinal barrier integrity, thus facilitating the transit of pathogenic microorganisms and their toxic metabolites from gut lumen to underlying layers (e.g., lamina propria, endowed with immune function) (Zhang et al. 2022; Bae et al. 2022; Mosiej et al. 2025). Recently, some probiotic bacteria showed to benefit host health through restoration of the intestinal barrier function (Mosiej et al. 2025). For instance, probiotic strains of *L. plantarum* group and *Bifidobacterium longum* subsp. *infantis* could restore the intestinal barrier by increasing the transcription level of genes involved in the TJs (Ashida et al. 2011; Wierzbicka et al. 2021; Wei et al. 2022; Zhang et al. 2022; Oudat and Okour 2025). Restoration of barrier function could also be obtained through administration of cell‐free supernatant (CFS) obtained from probiotic microorganisms, thus overlooking the “postbiotic” frontier (Tiptiri‐Kourpeti et al. 2016; Wierzbicka et al. 2021; Zhang et al. 2022; Dasriya et al. 2024). Furthermore, probiotics may display their beneficial activity also when administered as food ingredient (Nithya et al. 2023). However, in the latter case it is hard to define the borders between the food matrix and the probiotics, in terms of their contribution to the observed beneficial effect.

This study aims to understand the in vitro effect of two selected lactobacilli with probiotic traits on intestinal permeability and level of expression of OCLN, CLDN2, and LCN2. The lactobacilli were tested in different preparations: (i) cell biomass from single liquid culture; (ii) CFS from single liquid culture; (iii) zgougou obtained through the combination of the two lactobacilli as starters. In addition, spontaneously fermented zgougou was also subjected to the same assays, using human Caco‐2 cells monolayer, as in vitro model for intestinal epithelium.

## 2 Material and Methods

### 2.1 Preparation of Epithelial Cell Monolayers (Cell Culture)

Caco‐2 (Human Caucasian colon adenocarcinoma) cells, a cancer cell line of large intestine, purchased from ATCC (Rockville, USA), were seeded in the upper part of Transwell 12 well plates with 0.4 μm pore size (Corning Inc., New York, USA) containing Dulbecco Modified Eagles Medium (DMEM, Thermo Fisher Scientific, Massachusetts, USA) supplemented with L‐Glutamine, 10% heat‐inactivated Fetal Bovine Serum (Thermo Fisher Scientific), 1% of (1 M) 4‐(2‐hydroxyethyl)‐1‐piperazineethanesulfonic acid (HEPES, Sigma‐Aldrich, St. Louis, USA), 1% of sodium puryvate (100 mM) solution (Sigma‐Aldrich), 1% of streptomycin (10 mg/mL) solution and penicillin (10,000 U/mL) solution (Thermo Fisher Scientific). Cells were incubated at 37°C in a 5% CO_2_ atmosphere for 21 days, feeding them, on alternate days, with fresh DMEM. Full differentiation of monolayer was confirmed through measurement of transepithelial electrical resistance (TEER), using the Millicell ERS Voltohmmeter (Millipore, Eschborn, Germany) and visual control of cell layer integrity using a microscope, following the steps elsewhere reported (Huang 2009; Talà et al. 2022).

### 2.2 Preparation of Bacterial Cultures and Zgougou Juices

*Lactiplantibacillus paraplantarum* A1 and *L. plantarum* A2, previously isolated from traditional zgougou (Missaoui et al. 2019) and belonging to the Culture Collection of the Department of Soil, Plant, and Food Sciences, University of Bari ALDO MORO, Italy and the Department of Bio‐Resources: Integrative Biology & Valorization, Higher Institute of Biotechnology of Monastir, University of Monastir, Tunisia, were cultured in de Man Rogosa and Sharpe (MRS) broth (Oxoid, Basingstoke, Hamsphire, UK) at 37°C for 24 h, under anaerobic conditions. The culture broth was then centrifuged (10,000×*g*, 20 min, 4°C) and CFS was separated from cell biomass. Zgougou juice was obtained from a mixture of Aleppo pine ground seeds and tap water according to a ratio 2:3 (w:v), inoculated with *L. paraplantarum* A1 and *L. plantarum* A2 (J A1 + A2), both at cell density of 8 log CFU/g, and fermented at 25°C for 24 h, as previously detailed (Minervini et al. 2020). Additionally, juice from spontaneously fermented zgougou (J) was obtained after fermentation at 25°C for 24 h, without addition of lactobacilli. Both the zgougou started with selected lactobacilli and the zgougou obtained through spontaneous fermentation were stored at −20°C until analysis.

### 2.3 Exposure of Caco‐2 Cell Line to Bacterial Biomass, CFS, and Zgougou Juices

After their differentiation (21 days, value of TEER: about 193 Ω·cm^2^), Caco‐2 cells were transferred to new Transwell 12 well plates. When confluence was reached (about 6 days), cells were stimulated individually for 24 h with: biomass of probiotic bacteria A1 and A2 (both at cell density in the order of 8 log CFU/mL) cultured in MRS broth, CFS extracted from the same bacteria, spontaneously fermented zgougou juice (J), and zgougou juice fermented with probiotic bacteria A1 and A2 (J (A1 + A2)). An empty well, not containing cells, was used as the blank. Untreated cells, namely cells not exposed to any bacterial preparation or zgougogu juice, were used as negative controls in all experiments (vehicle). As a positive control 1 μg/mL of *Escherchia coli* lipopolysaccharide (LPS) (Sigma‐Aldrich) was used. Plates were then incubated at 37°C in 10% CO_2_ and 90% air. TEER was measured using a Millicell ERS Voltohmmeter and cell monolayer integrity was visually controlled under the microscope following the steps reported previously (Talà et al. 2022). The integrity of the monolayer was assessed after 10 min, 30 min, 1, 2, 6, and 24 h of incubation. Measurements were expressed in standard units (Ω·cm^2^) after subtracting mean values for resistance measured in the blank. Two technical replicates of biological triplicates were carried out.

### 2.4 Expression Level of Genes Related to Intestinal Barrier Integrity

The level of expression of genes related to intestinal barrier integrity was analyzed through RT‐qPCR. Total RNA was extracted from cells exposed for 24 h to the aforementioned treatments using the Invitrogen TRIzol Reagent (Thermo Fisher Scientific), following the manufacturer's instructions. Total RNA (500 ng) was reverse‐transcribed to cDNA using iScript cDNA Synthesis kit (Biorad, California, USA) with random primers, as described elsewhere (Parlesak et al. 2004; Talà et al. 2022). The following TaqMan (Thermo Fisher Scientific) gene expression assays were used: Hs00174128_m1for the *TNFα* gene, coding for tumor necrosis factor; Hs00252666_s1 for the *CLDN2* gene, coding for claudin 2; Hs05465837_g1 for the *OCLN* gene, coding for occludin; Hs01008571_m1 for the *LCN2* gene, coding for lipocalin 2; and Hs02758991_g1 for the *GAPDH* gene, coding for glyceraldehyde‐3‐phosphate dehydrogenase. The latter was used as the housekeeping gene. PCR was performed on a CFX96 System (Biorad), using the reaction mixture and conditions reported by Talà et al. (2022). The level of expression of the target genes was calculated using the ΔΔC_t_ method, with respect to the expression level of the *GAPDH* gene. Three technical replicates of biological triplicates were carried out.

### 2.5 Quantification of TNFα

The cell culture medium was recovered from the upper and lower part of the wells hosting the cells exposed to the different treatments and centrifuged (10 min). Supernatants were subjected to quantification of TNFα using the ELISA kit (RD Systems, Minneapolis, MN, USA) with 96‐well plates, as described by Bermudez‐Brito et al. (2015). Two technical replicates of biological triplicates were carried out.

### 2.6 Statistical Analysis

Statistical analysis was performed using Graphpad Prism 9.3.1. All data were expressed as means ± SD. Statistical significance were assessed with two‐tailed Student's *t*‐test and two‐way Anova test (*p < 0.05; **p < 0.01; ***p < 0.001; ****p < 0.0001). Results were considered statistically significant at *p* < 0.05.

## 3 Results and Discussions

### 3.1 Impact of Probiotics and Fermentation on Caco‐2 Cell Line Integrity

To the best of our knowledge, this is the first study that analyzed the influence on epithelial intestine cell integrity of two lactobacilli ( *L. paraplantarum* A1 and *L. plantarum* A2) isolated from zgougou (Missaoui et al. 2019), their CFS, zgougou juice fermented with the above lactobacilli (J (A1 + A2)) (Minervini et al. 2020), and zgougou juice spontaneously fermented (J). The analyses made use of Caco‐2 monolayer cells, as in vitro model of human gut epithelium.

We found that LPS (1 μg/mL) treatment caused a time‐dependent response in TEER values, compared to untreated cells (negative control). In detail, the TEER value of Caco‐2 monolayer after 30 min of exposure to LPS was significantly (*p* < 0.001) lower (ca. 160 Ω·cm^2^) than TEER of untreated cells; on the contrary, after 2 h of exposure to LPS, TEER increased to ca.193 Ω·cm^2^, being significantly (*p* < 0.05) higher than untreated cells (Figure 1). Lower TEER values directly correlate with increased permeability of the cell monolayer, indicating disruption of the intestinal barrier and onset of a “leaky” gut. This damage to the TJs can trigger the inflammatory pathways, leading to the release of pro‐inflammatory cytokines, as previously reported (Tunisi et al. 2019). Gut integrity is maintained by the epithelial junction adhesion complex, the mucous layer, and released products by both host cells and bacteria (Han et al. 2023).

**FIGURE 1:**
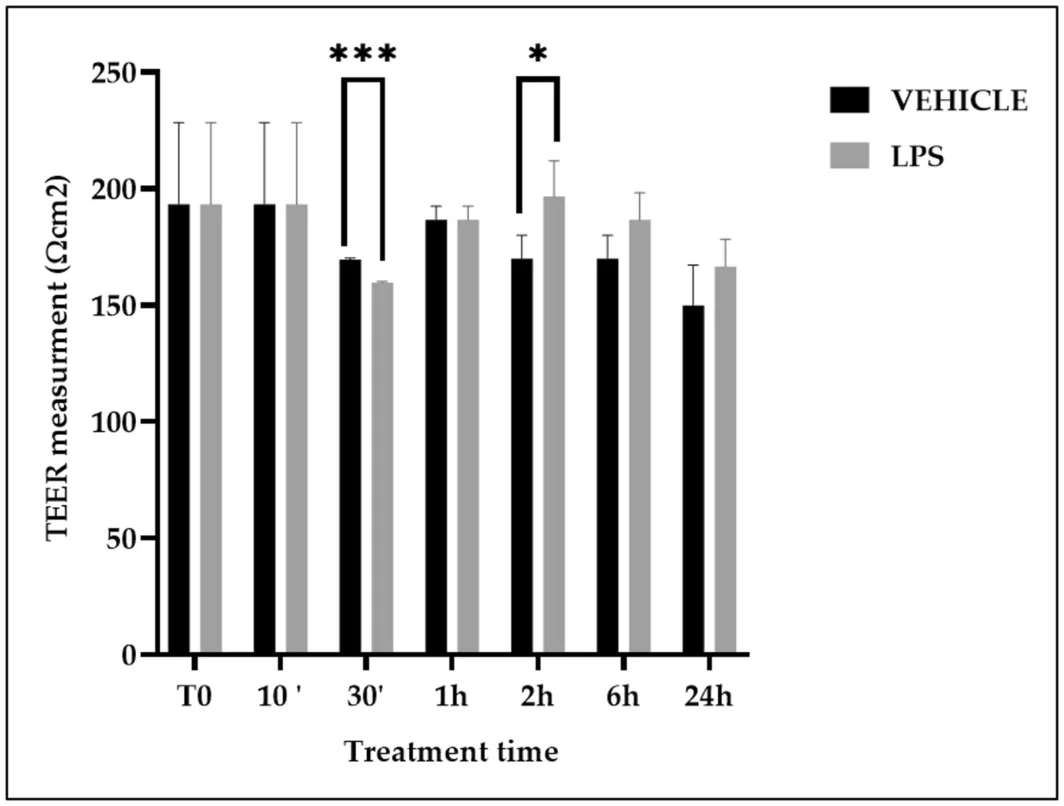
Average trans‐epithelial electrical resistance (TEER; Ω·cm^2^) measurements of Caco‐2 monolayer after 10′, 30′, 1 h, 2 h, 6 h and 24 h of treatment with LPS (positive control); (*p < 0.05; ***p < 0.001).

Caco‐2 monolayer exposed to *L. paraplantarum* A1 biomass diluted at 1:100 showed increased TEER values after 10 min (*p* < 0.0001) and 2 h (*p* < 0.05), compared to untreated cells; afterwards no significant (*p* > 0.05) differences were found (Figure 2A). When the monolayer was exposed to the biomass of the same LAB diluted (1:10), TEER increased after 30 min (*p* < 0.0001) and 24 h (*p* < 0.05). However, a decrease (*p* < 0.01) of TEER was strikingly found after 1 h with the latter sample (Figure 2A). When Caco‐2 monolayer was exposed for 6 h to the CFS of *L. paraplantarum* A1 (diluted 1:100), TEER was found significantly (*p* < 0.05) higher than the untreated cells (Figure 2B). Treatment of Caco‐2 monolayer with the biomass of *L. plantarum* A2 (diluted 1:100) resulted in significantly (*p* < 0.05) higher TEER (213 Ω·cm^2^) than the untreated cells only at 30 min (Figure 2C). At 30 min, TEER of the monolayer cells treated with the diluted (1:10) CFS of *L. plantarum* A2 was significantly higher than the untreated cells (Figure 2D). Overall, the treatment of Caco‐2 cell line by probiotic LAB could positively influence the cell barrier integrity, but depending on the LAB used, dose of LAB use and time of exposure. Probiotics, particularly those belonging to the *Lactiplantibacillus* genus, can improve gut health by several mechanisms, one of which could be the preservation of a good intestinal barrier (Dasriya et al. 2024; Mosiej et al. 2025). Those results are consistent with previous studies that showed positive effects on cell membrane integrity upon treatment with *L. plantarum* ; however, some strains were reported not to affect TEER values (Klingberg et al. 2005; Anderson et al. 2010; Tunisi et al. 2019; Dasriya et al. 2024; Mosiej et al. 2025). *L. plantarum* C06 and *Lactobacillus acidophilus* C11, isolated from pickled cabbage, showed good in vitro performance in terms of duodenal cell adhesion and gut biotic stress tolerance (Wang et al. 2010; Wang et al. 2015; Aslam et al. 2020; Wang et al. 2021). Under our experimental conditions, the increase of TEER observed after prolonged (30 min—6 h) exposure of Caco2 cell line to the CFS of both the lactobacilli would suggest that some bioactive molecules present in the CFS of lactobacilli may have crucial roles in cell–cell interaction, in concordance with previous reports (Dasriya et al. 2024; Mosiej et al. 2025).

**FIGURE 2:**
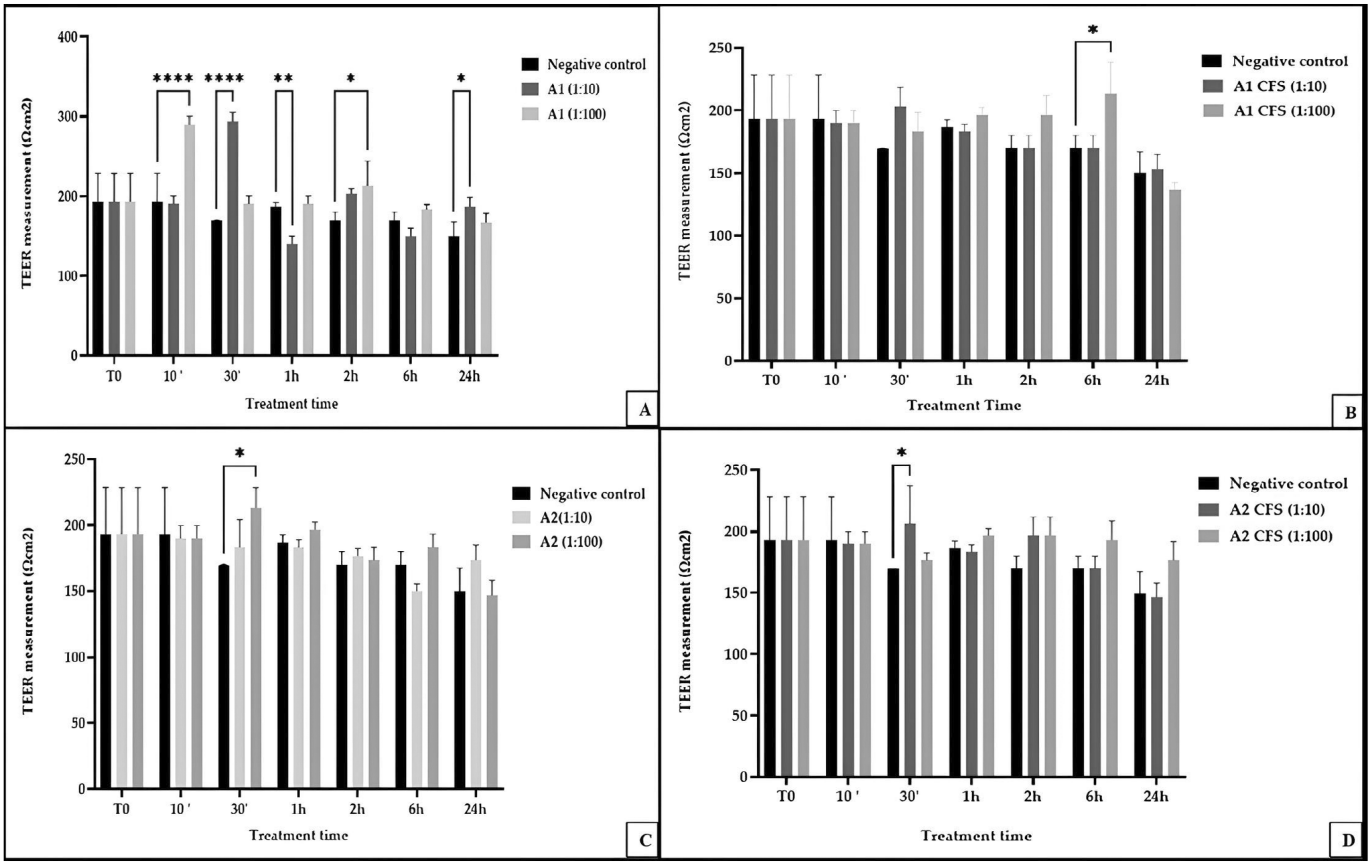
Average trans‐epithelial electrical resistance (TEER; Ω·cm^2^) measurements of Caco‐2 monolayer after 10′, 30′, 1 h, 2 h, 6 h and 24 h of treatment with *L. paraplantarum* cells A1 (1: 10) and (1: 100) (A); with A1 CFS (1: 10) and (1: 100) (B); *L. plantarum* cells A2 (1: 10) and (1: 100) (C) and with A2 CFS (1: 10) and (1: 100) (D). (*p < 0.05; **p < 0.01; ****p < 0.0001).

When the Caco‐2 monolayer was exposed to the zgougou juice fermented by *L. paraplantarum* A1 and *L. plantarum* A2 (J A1 + A2) and diluted 1:10, we found at all time‐points higher (*p* < 0.0001) values of TEER (690–963 Ω·cm^2^) than the negative control. No significant (*p* > 0.05) differences were observed when cells were treated with J A1 + A2 diluted 1:100 (Figure 3A). The only significant (*p* < 0.05) difference found during treatment of Caco‐2 monolayer with spontaneously fermented zgougou juice (J) was found after 30 min of exposure to J diluted 1:10, resulting in higher TEER than the negative control (Figure 3B). Our findings are in concordance with previous reports about plant‐based fermented food items which, being rich in microbes and their metabolites, in addition to their phytochemicals, can modulate the permeability of the intestinal barrier (Han et al. 2023). Different studies reported that fermented foods such as kefir, dahi, and korean kimchi, using different probiotic LAB strains, could be advantageous against many diseases such as cancer, IBD, anorexia nervosa, cardiovascular diseases, and diabetes (Şengün and Güney 2021; Nithya et al. 2023; Patel et al. 2023). Under our experimental conditions, we found that the two probiotic strains, although resulting in non‐univocal effect when singly tested, when used in combination to drive the zgougou juice fermentation could improve gut integrity in short time. Besides the effect related to the combination of probiotic lactobacilli and the vegetable matrix (Aleppo pine seeds) subjected to study, we could postulate that further interactions between the probiotic lactobacilli and host gut could occur upon ingestion. Indeed, vegetable matrices may protect probiotics during their passage through the gastrointestinal tract, until their subsequent release in the colon (Lavermicocca 2006; Han et al. 2023). Thus, these results underscore the potential effect of zgougou juice, fermented with selected lactobacilli, for the reinforcement of the intestinal epithelial barrier, but further investigations on the cell–cell interaction are required.

**FIGURE 3:**
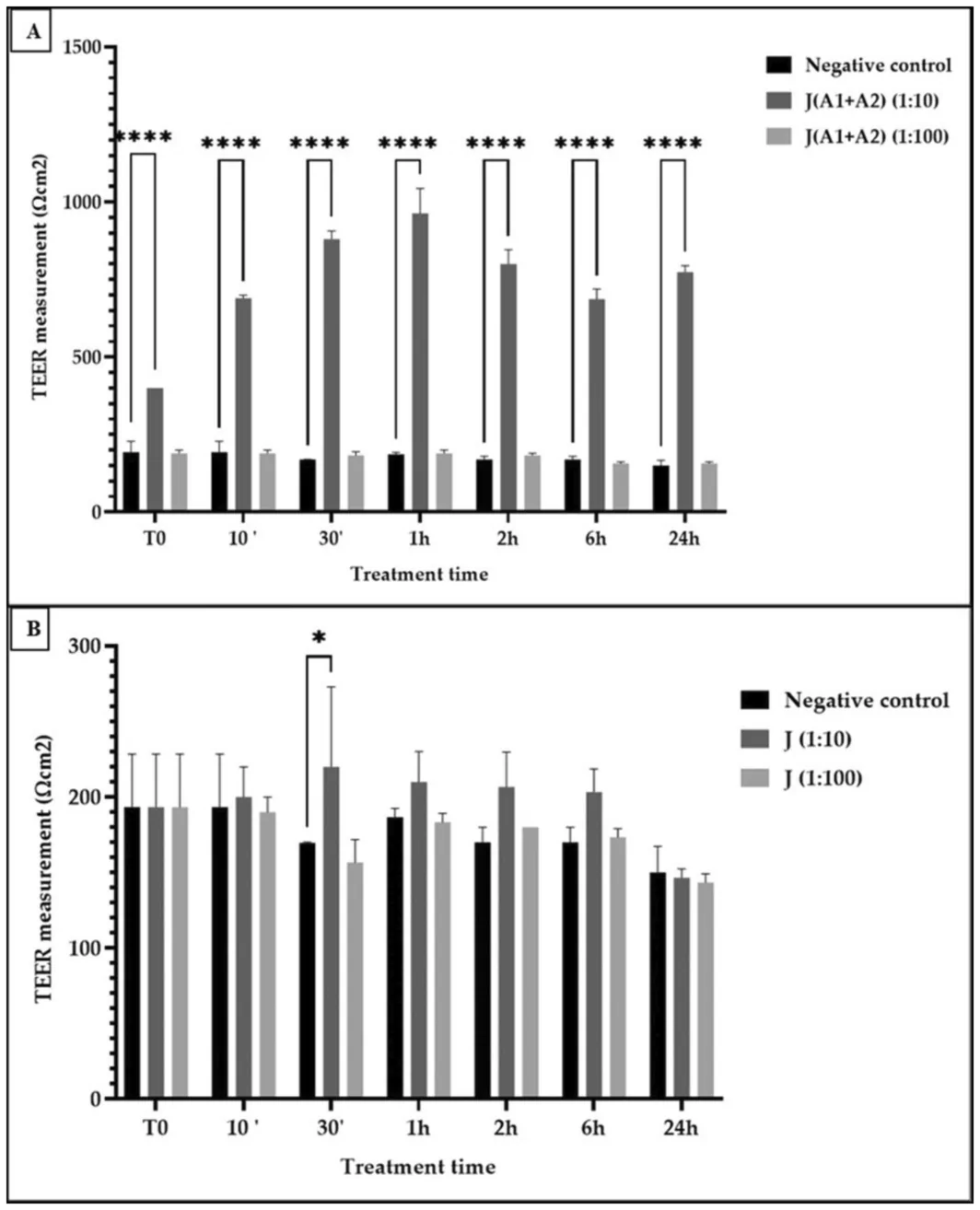
Average trans‐epithelial electrical resistance (TEER; Ω·cm^2^) measurements of Caco‐2 monolayer after 10′, 30′, 1 h, 2 h, 6 h and 24 h of treatment with fermented zgougou juice driven with LAB starter culture J (A1 + A2) at (1: 10) and (1: 100) (A) and zgougou juice spontaneously fermented J at (1: 10) and (1: 100) (B); (*p < 0.05; ****p < 0.0001).

### 3.2 Modulation of TNFα Gene Expression in Caco‐2 Monolayers

Cell biomasses of *L. paraplantarum* A1 and *L. plantarum* A2 significantly (*p* < 0.0001) increased the mRNA expression level of the gene coding for the pro‐inflammatory cytokine TNFα (Figure 4A). On the opposite, exposure of Caco‐2 cells to CFS from the aforesaid lactobacilli did not result in any significant variation of expression level of *TNFα* gene. In addition, the diluted zgougou juice fermented with both lactobacilli (J (A1 + A2)) strongly enhanced *TNFα* gene expression (*p* < 0.001) (Figure 4A). Spontaneously fermented zgougou juice (J) also increased *TNFα* expression, although with a milder effect than J (A1 + A2). TNFα is regarded as a pro‐inflammatory cytokine, but in some cases, it may be involved in promoting immune responses against pathogens. Gut microbiota can influence the expression of various immune‐related genes. Different LAB strains can have varying effects on TNF‐α production, highlighting the need for careful selection and characterization of probiotic strains. Probiotic LAB that inhibit or induce TNF‐α expression may have immune‐stimulating or immune‐suppressant effects, respectively (Yang et al. 2015; Saha et al. 2017; Bae et al. 2022; Sahu et al. 2023; Oladejo et al. 2024).

**FIGURE 4:**
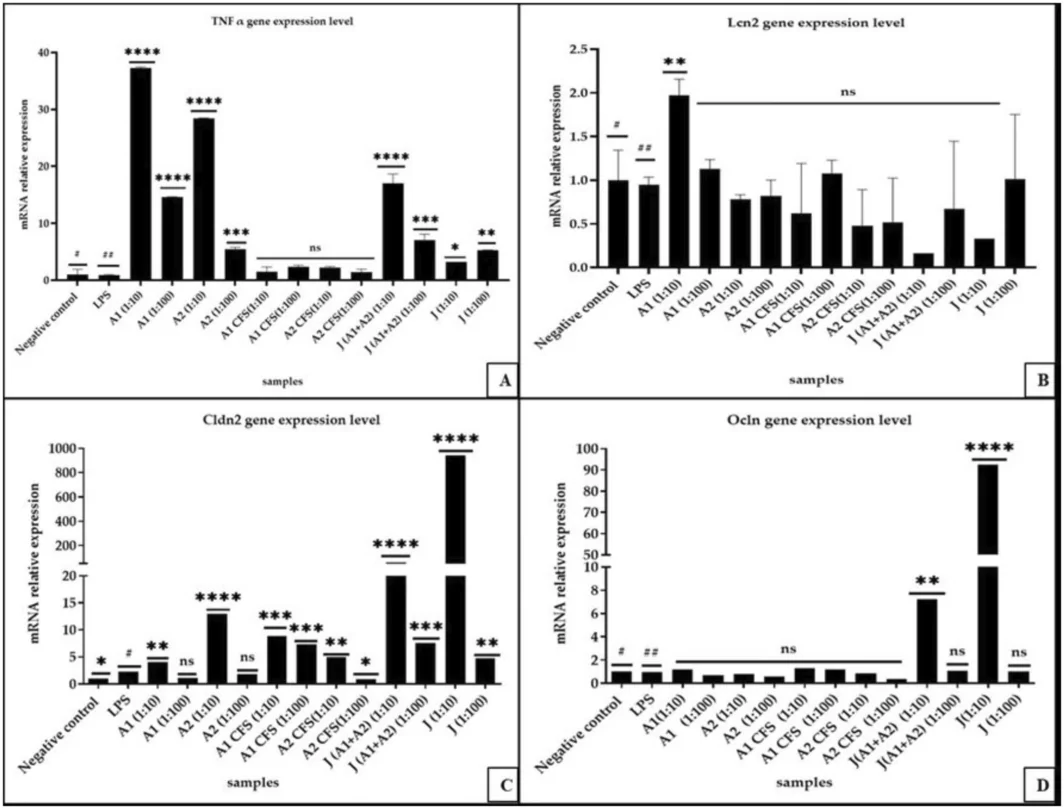
Differential mRNA levels of pro‐inflammatory cytokine and tight junctions' marker genes in Caco‐2 cells incubated with different samples as determined by RT‐qPCR after 24 h of treatment. (A) Normalized mRNA levels of pro‐inflammatory cytokine TNFα seeded on multiwell plates. (B) Normalized mRNA levels of LCN2 marker genes in Caco‐2 cells, (C) Normalized mRNA levels of Cldn 2 marker genes in Caco‐2 cells, (D) Normalized mRNA levels of Ocln marker genes in Caco‐2 cells which were seeded, respectively, on the top and the bottom of the Transwell system, after 24 h of incubation. #: negative control; ##: positive control; ns, no significance reported; *p < 0.05; **p < 0.01; ***p < 0.001; ****p < 0.0001.

Under our experimental conditions, although Caco‐2 cells exposed to either lactobacilli biomass or zgougou fermented with those lactobacilli showed higher expression of *TNF‐α* gene, ELISA assay did not detect differences of TNFα concentration between the upper and lower supernatant (Table S1). The discrepancy found between the level of expression of the gene coding for TNFα and no differences in the levels of TNFα could be explained by transcriptional and post‐transcriptional regulatory systems, which may have blocked the synthesis of this pro‐inflammatory cytokine (Dean et al. 2001; Chiewchengchol et al. 2015; Kobayashi et al. 2023).

### 3.3 Differential Expression of Genes Involved in Integrity of Intestinal Barrier

Caco‐2 monolayer exposed to *L. paraplantarum* A1 biomass (1:10 dilution) showed an increased (*p* < 0.01) level of expression of *LCN2* gene, compared to negative (untreated cells) and positive (cells exposed to LPS) controls (Figure 4B). In addition, exposure of Caco‐2 monolayer to *L. paraplantarum* A1 biomass (1:10 dilution) favored (*p* < 0.01) the transcription of *OCLN* gene (Figure 4C). The CFS of the same bacterial strain seemed to induce an even stronger expression (*p* < 0.001) of *OCLN* gene. We also found higher level of expression of both genes (*LCN2* and *OCLN*) in Caco‐2 cells exposed to biomass (diluted 1:10) (*p* < 0.0001) and CFS of *L. plantarum* A2 (Figure 4B,C). TJP, including CLDN and OCLN, contribute to the fundamental barrier function of intestinal epithelium, allowing the selective transport of solutes while preventing hazardous compounds and pathogenic bacteria from reaching the intestinal lumen (Colegio et al. 2003; Campbell et al. 2017; Suzuki 2020; Paradis et al. 2021). The results obtained in the present study are in agreement with previous research that showed that specific strains of *L. plantarum* may strengthen the barrier function of the intestinal epithelium, through regulating TJP (Anderson et al. 2010; Karczewski et al. 2010; Wei et al. 2022; Zhang et al. 2022; Fetarayani et al. 2024). However, since the observed effects, besides being strain‐dependent, are affected by host and gut microbiota, more research is required to better understand the influence of *L. plantarum* on TJP and related consequences for gastrointestinal health (Ashida et al. 2011; La Fata et al. 2018; Wierzbicka et al. 2021; Oudat and Okour 2025; Mosiej et al. 2025).

When Caco‐2 monolayer was treated with zgougou juice fermented with *L. paraplantarum* A1 and *L. plantarum* A2 (J A1 + A2) and diluted 1:10, significantly higher level of expression of *CLDN2* (Figure 4C) and *OCLN* (Figure 4D) genes was found. Exposure to spontaneously fermented zgougou juice (J), diluted 1:10, resulted in even higher (*p* < 0.0001) expression of both the genes (Figure 4C,D). These results are partly in accordance with those of TEER, suggesting an improved barrier function of intestinal epithelium. We found apparent discrepancy between expression of *CLDN2* and results of TEER. However, functional influence of the corresponding protein claudin‐2 depends on the overall structure and composition of the TJ complex rather than on the level of expression of one single gene. In addition, CLDN2 may have a dual function in epithelial physiology and illness, such as tissue remodeling and epithelial homeostasis (Wu et al. 2025). Under our experimental conditions, the overexpression of *CLDN2* would probably indicate TJ remodeling rather than increased intestinal permeability. First, this study showed that fermented zgougou juice could increase the level of expression of genes coding for TJP. Aleppo pine seeds, the raw matter used for zgougou, contain bioactive compounds with antioxidant and anti‐inflammatory activity, such as linoleic and α‐linolenic acid, flavonoids, and phenolics (Nasri and Triki 2004; Cheikh‐Rouhou et al. 2006; Missaoui et al. 2019; Aslam et al. 2020; Minervini et al. 2020; Han et al. 2023). We hypothesize that, under our experimental conditions, the effects observed on the expression level of genes coding for TJP may derive from a synergy between bioactive compounds from Aleppo pine seeds and fermentation with LAB. Untargeted metabolomics to be performed on zgougou fermented spontaneously or with selected lactobacilli, as well as on biomass and CFS of selected lactobacilli, would be useful to understand which compounds could have more probably caused the observed effects on gene expression of Caco‐2 monolayer.

## 4 Conclusion and Perspectives

This study showed that zgougou juice, depending on the type of fermentation (spontaneous or driven by two selected autochthonous lactobacilli), may strengthen integrity of the human Caco‐2 monolayer, as shown by increased TEER and level of expression of genes coding for TJP. This effect may be regarded as potentially beneficial to gut. Future research could focus on: (i) understanding whether this effect could derive from a synergy between bioactive compounds and microbiota of the zgougou matrix; (ii) assessing the biological activities of zgougou juice in an animal model.

## Author Contributions

**Jihen Missaoui:** conceptualization, investigation, writing – original draft. **Marina Liso:** investigation. **Mohamed Lataoui:** formal analysis. **Rosangela Limongelli:** investigation, writing – original draft. **Fabio Minervini:** writing – original draft, writing – review and editing, supervision. **Lotfi Achour:** supervision, formal analysis. **Maria De Angelis:** writing – review and editing, conceptualization.

## Funding

The authors have nothing to report.

## Conflicts of Interest

The authors declare no conflicts of interest.

## Supporting information


**Table S1:** Concentration of TNFα protein synthesized by Caco‐2 monolayer after 24 h of exposure to different treatments, assessed through ELISA method.

## Data Availability

All data supporting the findings of this study are available within the article.

## References

[fsn372387-bib-0001] Ait Atmane, S. , Z. Aksoylu Özbek , P. Günç Ergönül , and B. Khettal . 2021. “Valorization of *Pinus halepensis* Mill. Seed Oil: Physicochemical Characteristics, Bioactive Compounds, and Antioxidant Activity as Affected by Location and Extraction Method.” Journal of Food Processing and Preservation 45, no. 6: e15548. 10.1111/jfpp.15548.

[fsn372387-bib-0002] Ait Atmane, S. , M. B. Batır , Z. A. Özbek , et al. 2024. “Cold Pressed *Pinus halepensis* Mill. Seed Oil for Potential Health Applications: Analgesic, Anti‐Inflammatory Effects, and Assessment of Inflammatory Mediators by RT‐qPCR in Skin Wound Healing.” Journal of Ethnopharmacology 319: 117157. 10.1016/j.jep.2023.117157. 37696441

[fsn372387-bib-0003] Anderson, R. C. , A. L. Cookson , W. C. McNabb , et al. 2010. “ *Lactobacillus plantarum* MB452 Enhances the Function of the Intestinal Barrier by Increasing the Expression Levels of Genes Involved in Tight Junction Formation.” BMC Microbiology 10: 316. 10.1186/1471-2180-10-316. PMC3004893 21143932

[fsn372387-bib-0004] Ashida, N. , S. Yanagihara , T. Shinoda , and N. Yamamoto . 2011. “Characterization of Adhesive Molecule With Affinity to Caco‐2 Cells in *Lactobacillus acidophilus* by Proteome Analysis.” Journal of Bioscience and Bioengineering 112, no. 4: 333–337. 10.1016/j.jbiosc.2011.06.001. 21763196

[fsn372387-bib-0005] Aslam, H. , J. Green , F. N. Jacka , et al. 2020. “Fermented Foods, the Gut and Mental Health: A Mechanistic Overview With Implications for Depression and Anxiety.” Nutritional Neuroscience 23, no. 9: 659–671. 10.1080/1028415X.2018.1544332. 30415609

[fsn372387-bib-0006] Bae, W. Y. , W. H. Jung , S. L. Shin , et al. 2022. “Investigation of Immunostimulatory Effects of Heat‐Treated *Lactiplantibacillus plantarum* LM1004 and Its Underlying Molecular Mechanism.” Food Science of Animal Resources 42: 1031–1045. 10.5851/kosfa.2022.e50. PMC9647188 36415572

[fsn372387-bib-0007] Bermudez‐Brito, M. , S. Muñoz‐Quezada , C. Gómez‐Llorente , et al. 2015. “ *Lactobacillus paracasei* CNCM I‐4034 and Its Culture Supernatant Modulate Salmonella‐Induced Inflammation in a Novel Transwell Co‐Culture of Human Intestinal‐Like Dendritic and Caco‐2 Cells.” BMC Microbiology 15, no. 1: 79. 10.1186/s12866-015-0408-6. PMC5353866 25887178

[fsn372387-bib-0008] Botella, L. , and J. J. Diez . 2011. “Phylogenic Diversity of Fungal Endophytes in Spanish Stands of *Pinus halepensis* .” Fungal Diversity 47, no. 1: 9–18. 10.1007/s13225-010-0061-1.

[fsn372387-bib-0009] Campbell, H. K. , J. L. Maiers , and K. A. DeMali . 2017. “Interplay Between Tight Junctions & Adherens Junctions.” Experimental Cell Research 358, no. 1: 39–44. 10.1016/j.yexcr.2017.03.061. PMC5544570 28372972

[fsn372387-bib-0010] Cheikh‐Rouhou, S. , B. Hentati , S. Besbes , et al. 2006. “Chemical Composition and Lipid Fraction Characteristics of Aleppo Pine ( *Pinus halepensis* Mill.) Seeds Cultivated in Tunisia.” Food Science and Technology International 12: 407–415. 10.1177/1082013206069910.

[fsn372387-bib-0011] Chiewchengchol, D. , H. L. Wright , H. B. Thomas , et al. 2015. “Differential Changes in Gene Expression in Human Neutrophils Following TNF‐α Stimulation.” Immunology and Inflammation Diseases 4, no. 1: 35–44. 10.1002/iid3.90. PMC4768069 27042300

[fsn372387-bib-0012] Colegio, O. R. , C. Van Itallie , C. Rahner , and J. M. Anderson . 2003. “Claudin Extracellular Domains Determine Paracellular Charge Selectivity and Resistance.” American Journal of Physiology. Cell Physiology 284, no. 6: C1346–C1354. 10.1152/ajpcell.00547.2002. 12700140

[fsn372387-bib-0013] Dasriya, V. L. , M. Samtiya , S. Ranveer , et al. 2024. “Modulation of Gut Microbiota Through Probiotics and Dietary Interventions.” Journal of the Science of Food and Agriculture 104, no. 11: 6359–6375. 10.1002/jsfa.13370. 38334314

[fsn372387-bib-0014] Dean, J. L. , R. Wait , K. R. Mahtani , et al. 2001. “The 3′ Untranslated Region of Tumor Necrosis Factor Alpha mRNA Is a Target of the mRNA‐Stabilizing Factor HuR.” Molecular and Cellular Biology 21, no. 3: 721–730. 10.1128/MCB.21.3.721-730.2001. PMC86664 11154260

[fsn372387-bib-0015] Devi, M. B. , A. Bhattacharya , A. Kumar , et al. 2024. “Potential Probiotic Lactiplantibacillus Plantarum Strains Alleviate TNF‐α.” Cell Communication and Signaling 22, no. 1: 520. 10.1186/s12964-024-01900-7. PMC11514838 39468700

[fsn372387-bib-0016] Fetarayani, D. , G. Soegiarto , I. S. Surono , et al. 2024. “ *Lactiplantibacillus plantarum* IS‐10506 Enhances Tight Junction Integrity.” Indonesian Biomedical Journal 16, no. 2: 162–171. 10.18585/inabj.v16i2.2995.

[fsn372387-bib-0017] Han, H. , Y. You , S. Cha , et al. 2023. “Multi‐Species Probiotic Strain Mixture Enhances Intestinal Barrier Function.” Microorganisms 11: 656. 10.3390/microorganisms11030656. PMC10056128 36985228

[fsn372387-bib-0018] Huang, F. C. 2009. “Upregulation of Salmonella‐Induced IL‐6 Production in Caco‐2 Cells.” Mediators of Inflammation 2009: 103890. 10.1155/2009/103890. PMC2828125 20204057

[fsn372387-bib-0019] Kadri, N. , B. Khettal , Y. Aid , et al. 2015. “Physicochemical Characteristics of Pinus Seeds From North Algeria.” Food Chemistry 188: 184–192. 10.1016/j.foodchem.2015.04.138. 26041181

[fsn372387-bib-0020] Karczewski, J. , F. J. Troost , I. Konings , et al. 2010. “Regulation of Human Epithelial Tight Junction Proteins by *Lactobacillus plantarum* In Vivo.” American Journal of Physiology. Gastrointestinal and Liver Physiology 298, no. 6: G851–G859. 10.1152/ajpgi.00327.2009. 20224007

[fsn372387-bib-0021] Klingberg, T. D. , M. H. Pedersen , A. Cencic , and B. B. Budde . 2005. “Application of Transepithelial Electrical Resistance to Evaluate Probiotic Activity.” Applied and Environmental Microbiology 71, no. 11: 7528–7530. 10.1128/AEM.71.11.7528-7530.2005. PMC1287686 16269795

[fsn372387-bib-0022] Kobayashi, K. , J. Mochizuki , F. Yamazaki , and T. Sashihara . 2023. “Yogurt Starter Strains Ameliorate Intestinal Barrier Dysfunction.” Tissue Barriers 12, no. 1: 2184157. 10.1080/21688370.2023.2184157. PMC10832913 36852963

[fsn372387-bib-0023] La Fata, G. , P. Weber , and M. H. Mohajeri . 2018. “Probiotics and the Gut Immune System: Indirect Regulation.” Probiotics and Antimicrobial Proteins 10, no. 1: 11–21. 10.1007/s12602-017-9322-6. PMC5801397 28861741

[fsn372387-bib-0024] Lavermicocca, P. 2006. “Highlights on New Food Research.” Digestive and Liver Disease 38: S295–S299. 10.1016/S1590-8658(07)60014-0. 17259095

[fsn372387-bib-0025] Minervini, F. , J. Missaoui , G. Celano , et al. 2020. “Use of Autochthonous Lactobacilli to Increase the Safety of Zgougou.” Microorganisms 8, no. 1: 29. 10.3390/microorganisms8010029. PMC7023124 31877880

[fsn372387-bib-0026] Missaoui, J. , D. Saidane , R. Mzoughi , and F. Minervini . 2019. “Fermented Seeds (“Zgougou”) From Aleppo Pine.” Microorganisms 7, no. 12: 709. 10.3390/microorganisms7120709. PMC6958562 31861080

[fsn372387-bib-0027] Mosiej, W. , Z. Dorota , K. Marcin , et al. 2025. “The Impact of Food‐Derived LAB on Gut Microbiota Modulation.” International Journal of Psychophysiology 213: 113147. 10.1016/j.ijpsycho.2025.113147.

[fsn372387-bib-0028] Nasri, N. , and S. Triki . 2004. “Analyse des Lipides des Graines de Pins de Tunisie ( *Pinus halepensis* Mill. et *Pinus pinea* L.).” Rivista Italiana Delle Sostanze Grasse 81: 244–247.

[fsn372387-bib-0029] Nithya, A. , S. Misra , C. Panigrahi , et al. 2023. “Probiotic Potential of Fermented Foods in Non‐Communicable Diseases.” Food Chemistry Advances 3: 100381. 10.1016/j.focha.2023.100381.

[fsn372387-bib-0030] Oladejo, B. O. , G. T. Akingbade , C. F. Adeboboye , and S. B. Adeoye . 2024. “ *Lactobacillus* Species Modulates TNF‐α.” Microbe 2: 100050. 10.5114/bta.2023.125085.

[fsn372387-bib-0031] Oudat, Q. , and A. Okour . 2025. “The Role of Probiotics in Modulating Gut Microbiota and Metabolic Health for Weight Management.” Acta Microbiologica Hellenica 70: 5. 10.3390/amh70010005.

[fsn372387-bib-0032] Paradis, T. , H. Bègue , L. Basmaciyan , F. Dalle , and F. Bon . 2021. “Tight Junctions as a Key for Pathogens Invasion.” International Journal of Molecular Sciences 22, no. 5: 2506. 10.3390/ijms22052506. PMC7958858 33801524

[fsn372387-bib-0054] Parlesak, A. , D. Haller , S. Brinz , A. Baeuerlein , and C. Bode . 2004. “Modulation of Cytokine Release by Differentiated CACO‐2 Cells in a Compartmentalized Coculture Model with Mononuclear Leucocytes and Nonpathogenic Bacteria.” Scandinavian Journal of Immunology 60: 477–485. 10.1111/j.0300-9475.2004.01495.x. 15541040

[fsn372387-bib-0033] Patel, P. , K. Butani , A. Kumar , S. Singh , and B. G. Prajapati . 2023. “Effects of Fermented Food Consumption on Non‐Communicable Diseases.” Food 12: 687. 10.3390/foods12040687. PMC9956079 36832762

[fsn372387-bib-0034] Prasetyo, R. V. , I. Surono , N. A. Soemyarso , et al. 2020. “ *Lactobacillus plantarum* IS‐10506 Promotes Renal Tubular Regeneration.” Beneficial Microbes 11, no. 1: 59–66. 10.3920/BM2019.0036. 32066257

[fsn372387-bib-0035] Saha, P. , B. Chassaing , B. S. Yeoh , et al. 2017. “Ectopic Expression of Innate Immune Protein Lipocalin‐2.” Inflammatory Bowel Diseases 23, no. 7: 1120–1132. 10.1097/MIB.0000000000001134. PMC5469687 28445245

[fsn372387-bib-0036] Sahu, B. , L. M. Johnson , M. Sohrabi , et al. 2023. “Effects of Probiotics on Colitis‐Induced Exacerbation of AD.” International Journal of Molecular Sciences 24: 11551. 10.3390/ijms241411551. PMC10381012 37511312

[fsn372387-bib-0037] Schiller, G. 2014. “Therapeutic Use of Aleppo Pine ( *Pinus halepensis* Mill.).” In Medicinal and Aromatic Plants of the Middle‐East. Medicinal and Aromatic Plants of the World, edited by Z. Yaniv and N. Dudai , vol. 2. Springer. 10.1007/978-94-017-9276-9_11.

[fsn372387-bib-0038] Şengün, İ. Y. , and D. Güney . 2021. “Probiotic Potential of Fermented Foods and Their Effects on Immune System.” Turkish Journal of Agriculture ‐ Food Science and Technology 9, no. 10: 1744–1750. 10.24925/turjaf.v9i10.1744-1750.3880.

[fsn372387-bib-0039] Sharma, G. , A. Sharma , I. Kim , et al. 2024. “A Dietary Commensal Microbe Enhances Antitumor Immunity.” Nature Immunology 25, no. 5: 790–801. 10.1038/s41590-024-01816-x. 38664585

[fsn372387-bib-0040] Suzuki, T. 2020. “Regulation of the Intestinal Barrier by Nutrients.” Animal Science Journal 91: e13357. 10.1111/asj.13357. PMC7187240 32219956

[fsn372387-bib-0041] Talà, A. , F. Guerra , S. C. Resta , et al. 2022. “Phenotyping of Fecal Microbiota of Winnie, a Rodent Model of Spontaneous Chronic Colitis.” Inflammation 45, no. 6: 2477–2497. 10.1007/s10753-022-01706-0. PMC9646540 35732858

[fsn372387-bib-0042] Tiptiri‐Kourpeti, A. , K. Spyridopoulou , V. Santarmaki , et al. 2016. “ *Lactobacillus casei* Exerts Anti‐Proliferative Effects.” PLoS One 11, no. 2: e0147960. 10.1371/journal.pone.0147960. PMC4744000 26849051

[fsn372387-bib-0043] Tukan, S. K. , K. Al‐Ismail , R. Y. Ajo , and M. M. Al‐Dabbas . 2013. “Seeds and Seed Oil Compositions of Aleppo Pine Grown in Jordan.” Rivista Italiana Sostanze Grasse 90: 87–93.

[fsn372387-bib-0044] Tunisi, L. , N. Forte , A. C. Fernández‐Rilo , et al. 2019. “Orexin‐A Prevents LPS‐Induced Neuroinflammation.” Frontiers in Endocrinology 10: 219. 10.3389/fendo.2019.00219. PMC6467935 31024456

[fsn372387-bib-0045] Wang, C. Y. , P. R. Lin , C. C. Ng , and Y. T. Shyu . 2010. “Probiotic Properties of *Lactobacillus* Strains Isolated From the Feces of Breast‐Fed Infants and Taiwanese Pickled Cabbage.” Anaerobe 16, no. 6: 578–585. 10.1016/j.anaerobe.2010.10.003. 20951815

[fsn372387-bib-0046] Wang, I. K. , Y. Y. Wu , Y. F. Yang , et al. 2015. “The Effect of Probiotics on Serum Levels of Cytokine in PD Patients.” Beneficial Microbes 6: 423–430. 10.3920/BM2014.0088. 25609654

[fsn372387-bib-0047] Wang, I. K. , T. H. Yen , P. S. Hsieh , et al. 2021. “Effect of a Probiotic Combination in CKD Patients.” Frontiers in Nutrition 8: 661794. 10.3389/fnut.2021.661794. PMC8200566 34136518

[fsn372387-bib-0048] Wei, C. X. , J. H. Wu , Y. H. Huang , et al. 2022. “ *Lactobacillus plantarum* Improves LPS‐Induced Caco2 Barrier Damage.” PLoS One 17, no. 5: e0267831. 10.1371/journal.pone.0267831. PMC9154120 35639684

[fsn372387-bib-0049] Wierzbicka, A. , D. Ma'nkowska Wierzbicka , M. Mardas , et al. 2021. “Role of Probiotics in CRC Patients.” Nutrients 13: 1160. 10.3390/nu13041160.

[fsn372387-bib-0050] Wu, S. , J. Gao , Y. Han , et al. 2025. “Balancing Act: The Dual Role of Claudin‐2 in Disease.” Annals of the New York Academy of Sciences 1546: 75–89. 10.1111/nyas.15311. 40101185

[fsn372387-bib-0051] Yang, Y. , X. Zhao , M. H. Le , et al. 2015. “Reutericyclin Producing *Lactobacillus reuteri* Modulates Development of Fecal Microbiota in Weanling Pigs.” Frontiers in Microbiology 6: 762. 10.3389/fmicb.2015.00762. PMC4516970 26284047

[fsn372387-bib-0052] Zaroual, H. , F. Boughattas , and R. Karoui . 2019. “Traditional Foods in Maghreb: Production and Research Progress.” In Traditional Foods. Food Engineering Series, edited by M. Al‐Khusaibi , N. Al‐Habsi , and M. Shafiur Rahman . Springer. 10.1007/978-3-030-24620-4_4.

[fsn372387-bib-0053] Zhang, X. , Y. Li , C. Zhang , et al. 2022. “Postbiotics From *Lactobacillus plantarum* 1.0386 Ameliorate TJ Injury.” Food & Function 13, no. 21: 11008–11020. 10.1039/d2fo00001f. 36040437

