# Supplementary material for: Impact of Tunisian Fermented Zgougou and Related Lactobacilli on Caco‐2 Cell Barrier Integrity: Upregulation of Key Tight Junction Proteins
Source: Food Sci Nutr. 2026 Sep 27;14(10):e72387. doi: 10.1002/fsn3.72387 (PMC13617255; doi:10.1002/fsn3.72387)
Supplement: Supplementary file 1 [file FSN3-14-e72387-s001.docx]

| SAMPLE | Concentration of TNFα (pg/mL) |
| --- | --- |
| Blank | 0 |
| Negative control – Upper supernatant | 2.9 |
| LPS – Upper supernatant | 2.9 |
| Cells of *L. paraplantarum* A1 (1:10) – Upper supernatant | 2.9 |
| Cells of *L. paraplantarum* A1 (1:100) – Upper supernatant | 2.9 |
| Cells of *L. plantarum* A2 (1:10) – Upper supernatant | 2.9 |
| Cells of *L. plantarum* A2 (1:100) – Upper supernatant | 2.9 |
| CFS (1:10) of *L. paraplantarum* A1 – Upper supernatant | 0 |
| CFS (1:100) of *L. paraplantarum* A1 – Upper supernatant | 2.9 |
| CFS (1:10) of *L. plantarum* A2 – Upper supernatant | 2.9 |
| CFS (1:100) of *L. plantarum* A2 – Upper supernatant | 2.9 |
| 1:10 diluted zgougou juice fermented by A1 and A2 – Upper supernatant | 2.9 |
| 1:100 diluted zgougou juice fermented by A1 and A2 – Upper supernatant | 2.9 |
| 1:10 diluted zgougou juice spontaneously fermented – Upper supernatant | 0 |
| 1:100 diluted zgougou juice spontaneously fermented – Upper supernatant | 2.9 |
| Negative control – Lower supernatant | 2.9 |
| LPS – Lower supernatant | 2.9 |
| Cells of *L. paraplantarum* A1 (1:10) – Lower supernatant | 2.9 |
| Cells of *L. paraplantarum* A1 (1:100) – Lower supernatant | 2.9 |
| Cells of *L. plantarum* A2 (1:10) – Lower supernatant | 2.9 |
| Cells of *L. plantarum* A2 (1:100) – Lower supernatant | 2.9 |
| CFS (1:10) of *L. paraplantarum* A1 – Lower supernatant | 2.9 |
| CFS (1:100) of *L. paraplantarum* A1 – Lower supernatant | 2.9 |
| CFS (1:10) of *L. plantarum* A2 – Lower supernatant | 2.9 |
| CFS (1:100) of *L. plantarum* A2 – Lower supernatant | 2.9 |
| 1:10 diluted zgougou juice fermented by A1 and A2 – Lower supernatant | 2.9 |
| 1:100 diluted zgougou juice fermented by A1 and A2 – Lower supernatant | 2.9 |
| 1:10 diluted zgougou juice spontaneously fermented – Lower supernatant | 2.9 |
| 1:100 diluted zgougou juice spontaneously fermented – Lower supernatant | 2.9 |

**Supplementary Table 1.** Concentration of TNFα protein synthesized by Caco-2 monolayer after 24 h of exposure to different treatments, assessed through ELISA method.
